# Elevated audiovisual temporal interaction in patients with migraine without aura

**DOI:** 10.1186/1129-2377-15-44

**Published:** 2014-06-24

**Authors:** Weiping Yang, Bingqian Chu, Jiajia Yang, Yinghua Yu, Jinglong Wu, Shengyuan Yu

**Affiliations:** 1 Department of Psychology, Faculty of Education, Hubei University, Hubei, China; 2 Biomedical Engineering Laboratory, Graduate School of Natural Science and Technology, Okayama University, 3-1-1 Tsushima-Naka, 700-8530 Kitaku, Okayama, Japan; 3 Department of Neurology, Chinese PLA General Hospital, Fuxing Road 28, Beijing 100853, China; 4 Bio-robotics and System Laboratory, Beijing Institute of Technology, Beijing, China

**Keywords:** Audiovisual integration, Migraine, Attention, Hypersensitivity, Race model

## Abstract

**Background:**

Photophobia and phonophobia are the most prominent symptoms in patients with migraine without aura. Hypersensitivity to visual stimuli can lead to greater hypersensitivity to auditory stimuli, which suggests that the interaction between visual and auditory stimuli may play an important role in the pathogenesis of migraine. However, audiovisual temporal interactions in migraine have not been well studied. Therefore, our aim was to examine auditory and visual interactions in migraine.

**Methods:**

In this study, visual, auditory, and audiovisual stimuli with different temporal intervals between the visual and auditory stimuli were randomly presented to the left or right hemispace. During this time, the participants were asked to respond promptly to target stimuli. We used cumulative distribution functions to analyze the response times as a measure of audiovisual integration.

**Results:**

Our results showed that audiovisual integration was significantly elevated in the migraineurs compared with the normal controls (*p* < 0.05); however, audiovisual suppression was weaker in the migraineurs compared with the normal controls (*p* < 0.05).

**Conclusions:**

Our findings further objectively support the notion that migraineurs without aura are hypersensitive to external visual and auditory stimuli. Our study offers a new quantitative and objective method to evaluate hypersensitivity to audio-visual stimuli in patients with migraine.

## Background

Migraine is a common, disabling disorder that is highly prevalent in the general population [1,2]. Migraine without aura, which has no early unusual symptoms, is the most common form of migraine. Photophobia and phonophobia are the most prominent symptoms for patients with migraine without aura [3,4]. The intensities of photophobia and phonophobia correlate positively with the intensity of headache pain [5,6]. These findings demonstrate that the intensity of one migraine symptom is associated with the intensity of other migraine symptoms. Moreover, hypersensitivity to a unimodal stimulus may not be restricted to that stimulus, but it may also lead to the further elevation of hypersensitivities to other stimuli; for example, the exposure to light can lead to greater hypersensitivity to sound [7]. Therefore, it is inadequate to independently research only one migraine symptom. In daily life, most external information is received from vision and sound signals. Vision and sound signals are received separately and integrated in the human brain and, thus, provide a comprehensive understanding of the real world. Therefore, it is important to study integration across sensory modalities. Recently, Schwedt [7] illustrated the significance of interactions between the processing of signals for understanding migraine symptoms and their underlying mechanisms.

In healthy subjects, some studies that investigated cross-modal processing regarding vision and sound have shown that bimodal audiovisual stimuli can be discriminated or detected more accurately and faster compared with unimodal auditory or visual stimuli presented alone [8,9]. This facilitative effect is called “audiovisual integration”. Conversely, audiovisual suppression reflects the response to audiovisual stimuli that produce a significant decrease in the neuron’s activity as compared with the responses to unimodal stimuli [10]. Many studies of audiovisual interaction have investigated healthy subjects when visual and auditory stimuli were simultaneously presented [8,11,12]. However, the multisensory information may be temporally asynchronous in real life; for example, we first see lightning and then hear thunder. To adapt to the environment, the brain can integrate audiovisual information over a wide range of temporal gaps and correctly match auditory and visual signals [13,14]. In some neurons, combinations of auditory and visual stimuli delivered at specific intervals (50 and 150 ms) can produce greater responses. However, at longer intervals (200 and 300 ms), audiovisual stimulation either produces a reduction of a neuron’s response or no interaction [10]. Moreover, Talsma et al. [15] found that the processing of auditory and visual stimuli across specific temporal intervals was influenced by attention in a visual and auditory discrimination task using event-related potential (ERP). Their results showed that the attention effects on the right-hemisphere visual P1 were largest in the visual with auditory delayed by 50 ms condition. However, some studies found that attention to the unimodal visual or auditory signal is different in patients with migraine with normal controls. For the unimodal visual stimulus, migraineurs have a wider reactive field of activation compared with normal controls because of the sensitivity of the occipital cortex to light stimuli [16,17]. Furthermore, the heightened excitability of the visual cortex affects the top-down attentional control of the visual cortex in a visual spatial attention task [18]. For the unimodal auditory stimulus, Demarquay et al. [19] investigated migraineurs with a classic auditory habituation paradigm. Their results showed that the auditory orienting component (N1) was larger in migraineurs compared with normal controls, which suggests that automatic attention is increased in migraineurs. These findings suggested that attention is greater in patients with migraine compared with normal controls as a result of the hypersensitivity of migraineurs to an auditory or visual stimulus. Furthermore, some researchers have reported that attention could modulate audiovisual interaction processes, and that audiovisual integration was larger in the attended conditions compared with the unattented conditions [20]. Thus, in the conditions with attending visual and auditory signals, we predicted that the audiovisual integration elicited by audio-visual stimuli across temporal intervals in patients with migraine without aura would be greater or have a wider range of temporal gaps compared with normal controls.

To confirm our predictions, we designed a visual and auditory discrimination task that used conditions with attending visual and auditory signals. The stimuli contained visual, auditory and audio-visual stimuli across different temporal intervals, which were randomly presented with equal probability. Each stimulus type contained a standard and a target stimulus. The task of the subjects was to respond to the target stimuli. By comparing the audiovisual integration between the patients with migraine without aura and the normal controls, we examined whether the audiovisual temporal interaction of the patients with migraine without aura would be greater and have a wider range of temporal gaps compared with the normal controls.

## Methods

### Participants

Demographic information regarding the subjects is provided in Table 1. Twenty-one (17 females and 4 males) migraineurs had a mean age of 32.6 ± 5.4 years, and the mean age of the 21 normal controls (18 females and 3 males) was 30.1 ± 5.8 years; there was no significant difference in age between the groups (t = 1.432, *p* = 0.16). The migraine patients were recruited in a randomized sequence from the International Headache Center of the Department of Neurology of the Chinese PLA General Hospital. The diagnostic criteria for migraine without aura were based on the third edition (beta version) of the International Headache Classification (ICHD-3 beta) [21]. Their attack frequency was 2.14 ± 1.26 per month, and the attack duration was 7.89 ± 7.44 hours. The duration of the disease was 10.41 ± 6.96 years (Table 1). The patients did not use medication for a minimum of one week prior to the examination. The healthy participants were recruited by advertisement. The healthy participants were tested with the same batteries as the migraine patients to ensure that none of the healthy participants suffered actually suffers from migraine. The mini-mental state examination (MMSE) [22] and the Montreal cognitive assessment (MoCA) [23] were used to evaluate the participants’ cognitive functioning; there were no significant differences in the MMSE (t = 0.498, *p* = 0.624) or the MoCA (t = 1.76, *p* = 0.094) between the groups. The experimental protocol was approved by the ethics committees of Okayama University and the Chinese PLA General Hospital.

**Table 1:** Demographic and clinical information

	**Migraine patients**	**Normal controls**
Sample size, no.	21	21
Male/female	4/17	3/18
Age	32.6 ± 5.4	30.1 ± 5.8
MMSE score (out of 30)	29.0 ± 0.7	29.0 ± 1.0
MoCA (out of 30)	27.3 ± 1.2	28.4 ± 1.5
Attack frequency (per month)	2.14 ± 1.26	--
Attack duration (hour)	7.89 ± 7.44	--
Disease duration (year)	10.41 ± 6.96	--

Data are presented as the means ± the standard deviation. MMSE, Mini-Mental State Examination; MoCA, Montreal Cognitive Assessment.

### Stimuli and tasks

Stimulus presentation and response collection were accomplished using Presentation software (Neurobehavioral Systems Inc., Albany, California, USA). Unimodal visual stimuli, unimodal auditory stimuli and bimodal audiovisual stimuli were presented randomly to the left or right hemispace. Each stimulus type had two subtypes in total; the target stimulus and the task-irrelevant stimulus.

**Figure 1:**
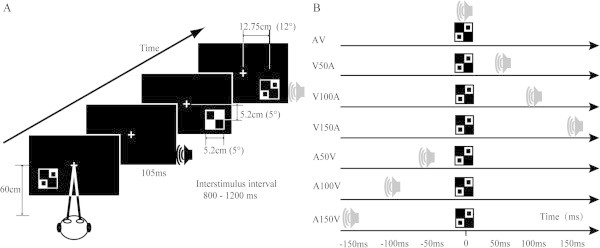
**Task. (A)** Subjects sat approximately 60 cm from the screen. The visual target stimulus was a checkerboard image with 2 dots that were contained within the checkerboard. The visual stimuli were randomly presented in the lower left or lower right quadrant of the screen. The auditory target stimulus was white noise (gray speaker). The auditory stimuli were presented to the left or right ear through earphones. The audiovisual target stimulus consisted of the simultaneous presentation of visual and auditory target stimuli. Each trial was randomly presented to the left or right of the central fixation point. **(B)** Decomposition of the relative timings of the auditory and visual stimuli within each subtype of audiovisual stimuli. AV, visual with simultaneous auditory; V50A, visual with auditory delayed by 50 ms; V100A, visual with auditory delayed by 100 ms; V150A, visual with auditory delayed by 150 ms; A50V, auditory with visual delayed by 50 ms; A100V, auditory with visual delayed by 100 ms; A150V, auditory with visual delayed by 150 ms.

The visual target stimulus was a checkerboard image with two black dots contained within the checkerboard (5.2 × 5.2 cm, subtending a visual angle of 5-degrees); it was presented on a black background on a 21-inch computer monitor positioned 60 cm in front of the participant’s eyes (Figure 1A). These visual stimuli were randomly presented to the lower left or lower right quadrant of the screen (at a 12-degree visual angle to the left or right of the center and a 5-degree angle below the central fixation) [20,24]. The auditory target stimulus consisted of white noise at 60 dB. The auditory stimuli were presented to the left or right ear through earphones. The audiovisual target stimuli were presented in the following multisensory conditions: simultaneous visual with auditory (AV); visual with auditory delayed by 50 ms, 100 ms or 150 ms (V50A, V100A and V150A, respectively); or auditory with visual delayed by 50 ms, 100 ms, and 150 ms (A50V, A100V and A150V, respectively; Figure 1B). Additionally, task-irrelevant stimuli were included to prevent the participants from habituating or predicting the target stimuli and enabled the subjects to be more attentive to the stimuli. The task-irrelevant stimuli composed 20% of the total stimuli [25,26]. The task-irrelevant visual stimulus was a checkerboard. The task-irrelevant auditory stimulus was a 1000 Hz sinusoidal tone and amplitude of 60 dB. The task-irrelevant audiovisual stimulus was composed of the task-irrelevant visual and auditory stimuli. The duration of each component of each stimulus was 105 ms.

In this experiment, 60 unimodal visual stimuli and 60 unimodal auditory stimuli were presented. For the audiovisual stimuli, 420 stimuli were presented; these stimuli included all audiovisual temporal asynchronies. All stimuli were presented with a randomly varied inter-stimulus interval (ISI; measured from the offset of one trial to the set of the next) between 800 and 1200 ms (mean = 1000 ms). During the ISI and unimodal auditory events, a fixation cross was centrally presented. On average, 5 blocks with duration of approximately 5 minutes each were presented. Each block began with a 3000 ms fixation period that was followed by the test stimuli. The participants were instructed to indicate whether the targets appeared on the left or right hemispace as quickly and accurately as possible.

### Data processing and analysis

#### *Response times and hit rates*

The response time was measured by hitting a button for the first stimulus presented. The response times were first analyzed to remove outliers, which were defined as responses that occurred faster or slower than 3 standard deviations from the mean response time for each subject. The hit rates were defined as the number of correct responses to target stimuli divided by the total target stimuli. Hit rates and response times were subjected to repeated-measures analyses of variance (ANOVAs). These analyses employed stimulus modality as the within-participants factor (nine levels: visual, auditory, and seven audiovisual levels). For all analyses, group (two levels: migraine patients and normal controls) served as the between-participants factor. The Greenhouse-Geisser Epsilon correction was applied to adjust the degrees of freedom of the F ratios as necessary.

#### *Independent race model*

In the analyses previously described, only a single central tendency score (i.e., the mean) was used to determine the response times. To control for the redundant nature of the audiovisual stimuli conditions, we used an independent race model [27,28]. This model analyzed the response times using cumulative distribution functions (CDFs). Audiovisual data were compared by evaluating the statistical facilitation using the CDF of the summed probability of the visual and auditory responses. To complete this analysis, the CDFs for each trial type were generated for each participant using 10-ms time bins. Each participant’s unimodal CDFs were used to calculate the race distribution at each time bin using the following formula: [P (A) + P (V)] – [P (A) × P (V)], where P (A) is the probability of responding within a given time in a unimodal auditory trial, and P (V) is the probability of responding within a given time in a unimodal visual trial. If the probability of the response to the bimodal audiovisual stimulus was significantly greater than the response predicted by the summed probability of the unimodal stimuli, audiovisual integration was considered to have occurred [27,28].

Each participant’s race model curves were then subtracted from their audiovisual CDFs that were generated for the different temporal intervals. A one-sample *t*-test was performed at each time bin within each group (patients with migraine without aura and normal controls) to compare these difference curves to zero, and significant (*p* < 0.05) deviations were identified [29,30]. Additionally, to obtain a measure of integration that was not affected by timing differences across individuals, the areas under each participant’s 7 difference curves were computed. The area under the curve was determined by computing the integral of the significant deviations region under each curve [31]. These area values were subjected to repeated-measures ANOVA, and group differences in integration were further assessed. All statistical analyses were performed using SPSS version 16.0 software (SPSS, Tokyo, Japan).

## Results

### Hit rates

The overall hit rates were greater than 80% for both the migraine and normal control groups, as shown in Table 2. A group (two groups) × condition (nine conditions) repeated-measures ANOVA revealed no main effect of group [F (1, 40) = 0.85, *p* = 0.362] and no significant interaction between group and stimulus condition [F (8, 320) = 1.248, *p* = 0.293].

**Table 2:** Mean response times and hit rates for normal controls and migraine patients

**Stimulus conditions**	**Response times (ms) (*P*** **= 0.1)**		**Hit rates (%) (*P*** **= 0.362)**	
	**Normal controls**	**Migraine patients**	**Normal controls**	**Migraine patients**
A	509 ± 14.7	550 ± 13.1	90 ± 1.4	89 ± 3.9
V	433 ± 11.2	468 ± 16.2	92 ± 2.3	93 ± 2.6
AV	361 ± 7.9	382 ± 12.6	96 ± 0.7	98 ± 0.6
V50A	389 ± 8.7	411 ± 11.6	96 ± 0.9	97 ± 1.1
V100A	399 ± 8.8	422 ± 12.8	95 ± 1.0	97 ± 1.3
V150A	415 ± 8.6	438 ± 12.9	95 ± 1.2	98 ± 1.0
A50V	384 ± 7.8	406 ± 11.2	96 ± 0.7	98 ± 0.9
A100V	412 ± 7.6	435 ± 11.6	96 ± 0.8	97 ± 1.2
A150V	432 ± 8.2	455 ± 12.1	95 ± 1.0	98 ± 1.3

Data are presented as the means ± the standard error of the mean (SEM). *P* values represent the main effects for group in response times and hit rates.

### Response times

The mean response times are shown in Table 2. A group (two groups) × condition (nine conditions) repeated-measures ANOVA was performed on the mean response times. The repeated-measures ANOVA revealed a significant main effect of stimulus condition [F (8, 320) = 199.0, *p* < 0.001]. However, no main effect was identified for group [F (1, 40) = 2.84, *p* = 0.1], and no significant interaction was found between group and stimulus condition [F (8, 320) = 1.13, *p* = 0.33]. In addition, an ANOVA was performed on the response times for the patients with ictal and interictal migraine status. The results showed that no significant interaction was found between ictal and interictal status [F (8, 152) = 0.395, *p* = 0.748]. Because of 8 patients with phonosensitivity and 7 patients with both photosensitivity and phonosensitivity, we investigated the reaction times to the stimuli between only phonosensitivity and both photosensitivity and phonosensitivity. No significant interaction was found for the groups [F (8, 104) = 2.407, *p* = 0.087].

### Race model comparisons

Race model comparisons of the response times of the migraine patients and the normal controls are discussed separately below.

#### *Simultaneous visual with auditory (AV)*

**Figure 2:**
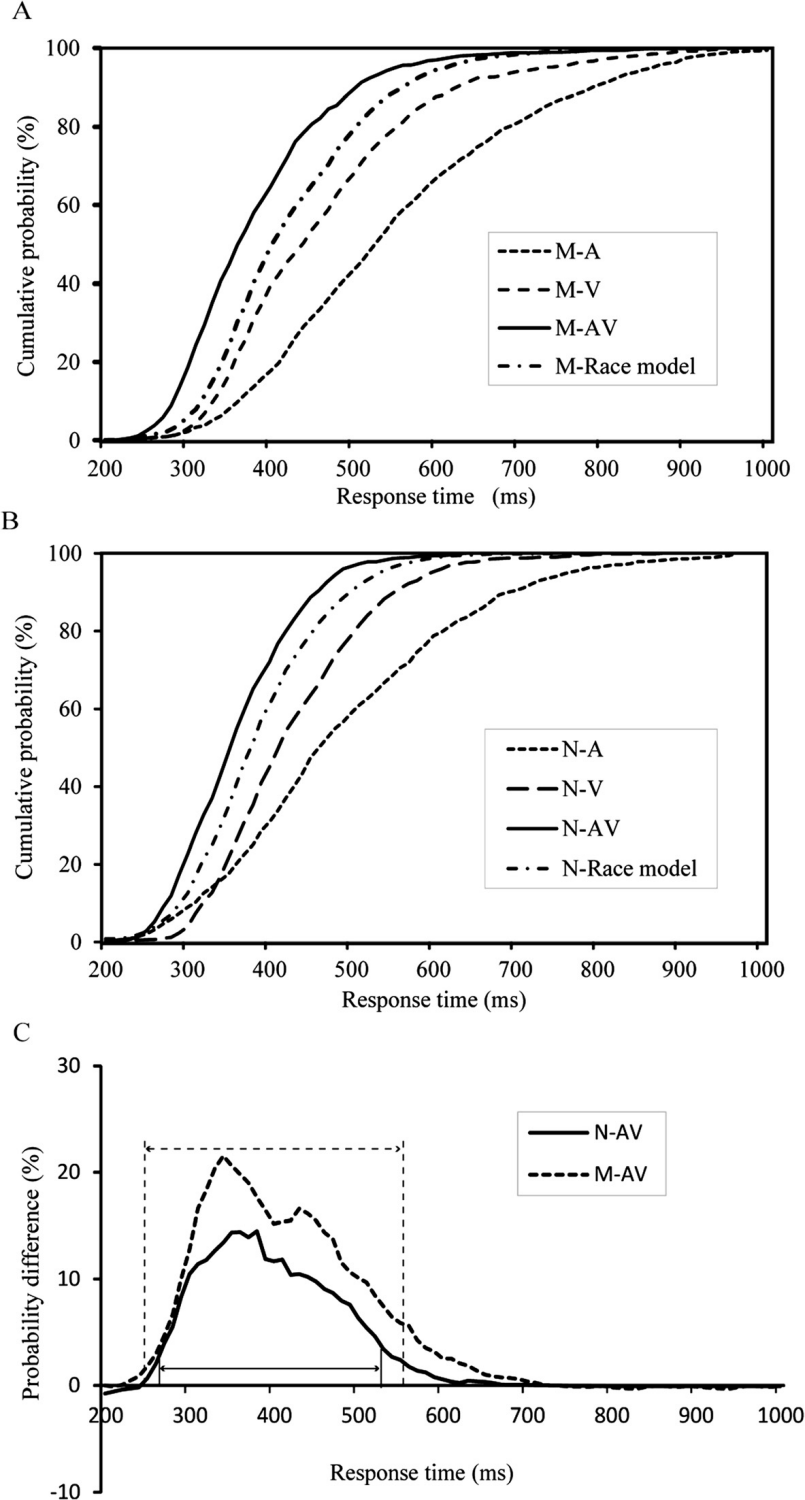
**Distributions of response times under in the visual with simultaneous auditory (AV) condition. (A)** Cumulative distribution functions (CDFs) for the discrimination response times to auditory (A), visual (V), and audiovisual (AV) stimuli in migraine patients (M). The summed probability of visual and auditory responses is shown by the race model curve (race model). Note that the audiovisual responses were typically faster compared with the race model predictions. **(B)** CDFs for normal controls (N). **(C)** The cumulative probability difference curves show the behavioral facilitations compared with the race model predictions for the migraine patients (dotted line, from 250 to 560 ms) and the normal controls (solid line, from 270 to 530 ms).

The CDFs of the response times to the visual, auditory, and AV stimuli are shown in Figure 2. A comparison of the CDFs clearly indicated that the responses to the bimodal AV stimuli were faster than those to the unimodal visual or auditory stimuli in both groups. Moreover, bimodal performance exceeded that predicted by the race model, which suggests that AV integration occurred. These relationships can easily be compared across the two groups by subtracting the race model CDF from the bimodal AV CDF for each group (Figure 2C). One-sample t-tests were performed on the resultant distributions to determine whether the multisensory facilitations were significantly above zero. Significant facilitations were found for the normal controls, with response times that ranged from 270 to 530 ms (*p* < 0.05) and peaked at 380 ms (14.51%), whereas the migraine patients exhibited extended response times (250 to 560 ms) that peaked later (340 ms, 21.56%).

#### *The visual and auditory stimuli were presented at a 50 ms temporal interval*

**Figure 3:**
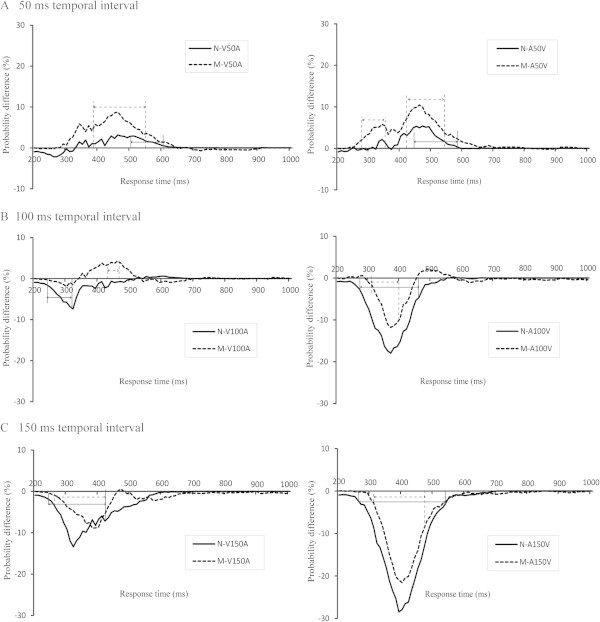
**The cumulative probability difference curves show the audiovisual interaction. (A)** The 50 ms; **(B)** 100 ms; **(C)** 150 ms temporal interval between the visual and auditory stimuli conditions for the migraine patients (dotted line) and the normal controls (solid line).

The same comparison that was performed for the AV condition was performed for the V50A condition. For the migraine patients, a significant elevation of integration (*p* < 0.05) occurred from 390 to 550 ms (with a peak of 8.75% at 460 ms), whereas the normal controls exhibited delayed response times that ranged from 510 to 610 ms (with a peak of 3.11% at 510 ms) (Figure 3A, left side). For the A50V condition, an elevation of integration (*p* < 0.05) in the migraine patients was found from 280 to 350 ms and from 420 to 530 ms (with a peak of 11.96% at 460 ms). The normal controls showed multisensory elevation from 440 to 590 ms (with a peak of 5.39% at 470 ms) (Figure 3A, right side).

#### *The 100 ms temporal interval between the visual and auditory stimuli conditions*

The response time distributions in the V100A condition were counted. A comparison of the CDFs of the bimodal V100A condition and the CDFs predicted by the statistical summation of the unimodal visual and auditory stimuli revealed a significant elevation in integration (*p* < 0.05) from 430 to 470 ms (with a peak of 4.43% at 460 ms) for the migraine patients. In contrast, a significant response suppression from 240 to 320 ms (with a peak of -8.63% at 320 ms) was found for the normal controls (Figure 3B, left side). In the A100V condition, significant behavioral suppressions were found in both the migraine patients (from 310 to 400 ms with a peak of -11.81% at 370 ms) and the normal controls (from 280 to 460 ms with a peak of -18.93% at 370 ms) (Figure 3B, right side).

#### *In the V150A and A150V conditions*

In these two conditions, the temporal intervals between the visual and auditory stimuli were 150 ms; hence, elevated audiovisual integration was not present in a series of analyses for the migraine patients or the normal controls (Figure 3C). Multisensory suppression in the migraine patients was observed from 270 to 420 ms with a peak of -8.93% at 380 ms and from 310 to 480 ms with a peak of -21.15% at 400 ms under the V150A and A150V conditions, respectively. The normal controls exhibited evidence of multisensory suppression from 250 to 420 ms with a peak of -13.07% at 320 ms and from 260 to 530 ms with a peak of -27.99% at 390 ms under the V150A and A150V conditions, respectively.

#### *Area under the curve*

**Figure 4:**
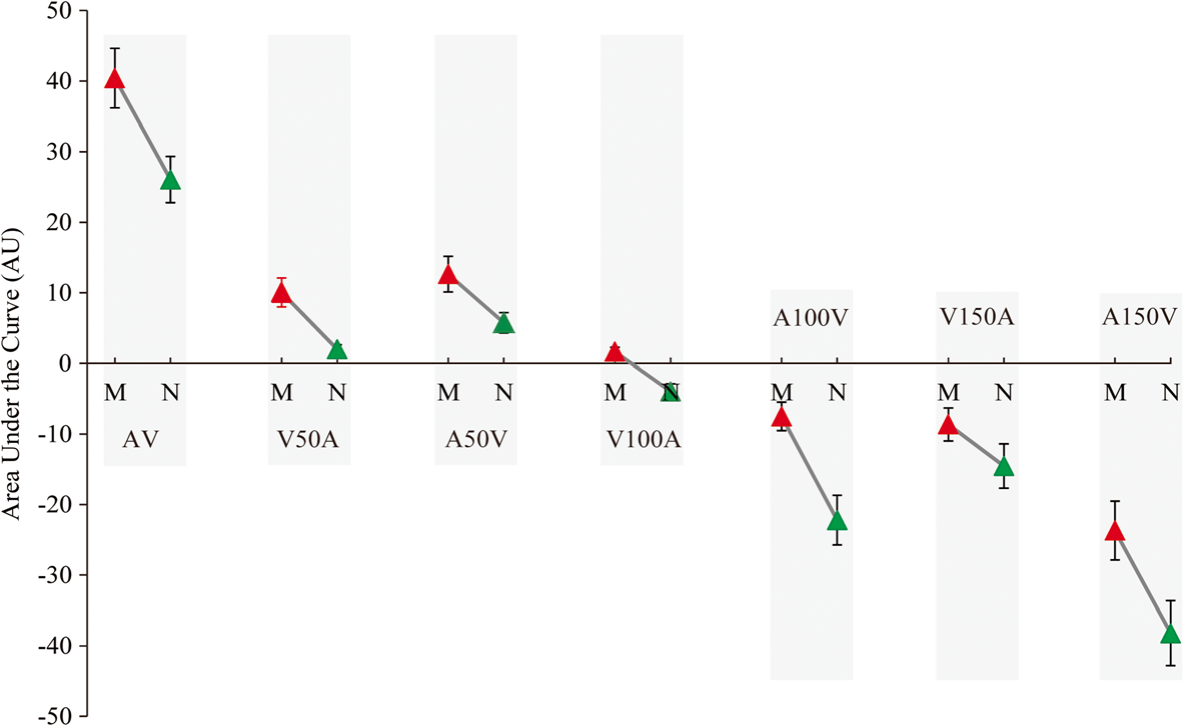
**The areas under the curves for the migraine patients (M) and the normal controls (N).** Experimental conditions are plotted on the x-axis. The areas under the curves are plotted on the y-axis.

Because each subject has a different time course of responses, averaging difference curves may not provide a complete indication of group differences. Areas under the curve for significant time periods were calculated for each subject to avoid timing differences. The group differences in integration were assessed using Group (two groups) × condition (seven conditions) repeated-measures ANOVA. Main effects of both group [F (1, 40) = 12.19, *p* = 0.001] and stimulus condition [F (6, 240) = 168.86, *p* < 0.001] were observed, however, no significant interaction was found between group and stimulus condition [F (6, 240) = 1.791, *p* < 0.143]. Thus, the areas under the curve were significantly different between the patients with migraine without aura and the normal controls as shown in Figure 4. These results showed that audiovisual integration was significantly elevated in the migraineurs compared with the normal controls, whereas, audiovisual suppression was weaker in the migraineurs compared with in the normal controls.

## Discussion

The main result of the present study is that the elevations in audiovisual integration were significantly greater in the migraineurs compared with the normal controls (*p* < 0.05).

### Audiovisual elevation

In the present study, the elevation of audiovisual integration in the migraineurs was significantly greater compared with the normal controls in the AV, V50A and A50V conditions (Figures 2 and 3A). These results are similar to those obtained in previous aging effect studies [29]. These previous studies examined the speed of discrimination responses to the presentation of visual, auditory or combined audiovisual stimuli in old and young individuals. The results of these studied revealed that older adults exhibited significantly greater multisensory audiovisual enhancement than younger adults. These findings suggest that the audiovisual elevation effects observed in older adults and migraine patients are similar. In older adults, cognitive ability regarding vision or sound decreases gradually with aging. The elevated audiovisual integration indicated that multiple sensory channels could supplement the unimodal sensory deficits that are associated with aging and suggested that the increase in audiovisual integration in elderly adults might be because of changes in multisensory processing. Further, the activities of the inferior parietal and medial prefrontal regions 100 ms after stimulus onset were increased in older compared with younger adults [32]. For the patients with migraine without aura, hypersensitivity to light or sound, which was different from healthy young individuals, might lead to the elevated audiovisual integration. However, further studies are needed to elucidate the neural mechanisms of the patients with migraine without aura. The elevated audiovisual integration in patients with migraine without aura and elderly adults might be driven by different mechanisms.

Another potential explanation for audiovisual elevation may be the top-down attention regulation of multisensory interactions. Previous research has indicated that attention modulates interactions between modalities [25,33]. The findings of these studies demonstrated that significant increases in audiovisual integration occur when attention is directed to visual and auditory modalities simultaneously, compared with when attention is focused on one modality alone [25,33]. Further audiovisual integration is greater for attended compared with unattended auditory and visual signals [20]. Previous studies have also shown that migraineurs have lower sound [3] and light [34,35] discomfort thresholds compared with normal controls. These findings further indicate that migraine patients are sensitive to visual and auditory stimuli and suggest that the same auditory and visual stimuli could naturally elicit more attention from migraineurs compared with normal controls.

In the V100A condition, an elevation of audiovisual integration was found in migraineurs, whereas audiovisual integration was suppressed in normal controls in this condition (Figure 3). These results indicated that migraineurs can integrate audiovisual information over a wider range of temporal intervals and correctly match sound and visual signals. Indeed, multisensory processing can not only affect established multisensory convergence zones, but it can also affect brain areas and responses that have traditionally been considered sensory specific (e.g., visual areas) [36,37]. Several neuroimaging studies have shown that migraineurs exhibit greater activation of the visual cortex and motion sensitive temporal cortical regions and exhibit atypical responses to visual stimulation in visual motion processing areas [38-40]. Such increases in neural responses in unimodal sensory areas have the potential to drive audiovisual information over a wider range of temporal gaps and result in feed-forward multisensory integration [37,41]. Additionally, the mechanical transduction of sound waves through the ear takes less time compared with the chemical transduction of light through the retinas [42,43]. Thus, the temporal difference between the two signals decreases in convergent brain areas when visual stimuli precede auditory signals by 100 ms. Furthermore, previous studies have found that the timing of auditory and visual stimuli affects integration [44]. These studies have also confirmed that integration is greater when the auditory stimuli are presented in close temporal proximity to the visual stimuli [45,46]. These results agree with the findings of the current study that audiovisual integration was elevated in the V100A condition, but not in the A100V condition.

### Audiovisual suppression

In this study, audiovisual suppression was weaker for the migraineurs compared with the normal controls. In the human perceptual system, attention to a single sensory modality acts to enhance neural activity related to that modality and suppress neural activity related to the unattended sensory modalities. Several studies have shown that the cerebral cortices of patients with migraine exhibit hyperexcitability to various external stimuli [47]. This cortical neuronal hyperexcitability may be a result of decreased activation of inhibitory systems in patients with migraine [48]. Moreover, migraineurs have been shown to have extraneous visual noise [49] and a reduced ability to suppress sensory-evoked activity [18]. Inefficient suppression may lead to weak audiovisual suppression in migraineurs compared with normal controls.

In the clinic, migraine diagnostic methods are predominately based on questions from physicians, which can be affected by the subjective experiences of the patients and physicians. Because the simple visual and auditory signal paradigms utilized in this study can be administered quickly and easily, they may provide an objective tool to estimate cognitive competence.

### Limitation of the current study

The limitations of the current research are the following: First, only patients with migraine without aura were investigated. Because of this limitation, patients with migraine could be not studied systematically. Second, only a simple behavioral analysis was implemented in the current study. Therefore, it is not possible to elaborate on the brain mechanisms of patients with migraine without aura because of the lack of imaging studies. In future studies, we would further research on patients with migraine including other migraine types. Furthermore, the pathophysiology of patients with migraine might be explained using simultaneous brain imaging and electrophysiological evidence.

## Conclusions

In this study, we used the race model as a tool to describe entire reaction time distributions rather than single central tendency scores. The data clearly showed that the elevations in audiovisual integration were significantly greater in the migraineurs than they were in the normal controls (*p* < 0.05). These findings suggest that elevated audiovisual integration may have a greater compensatory influence on unimodal sensory cognitive impairment. These results further objectively support the notion that migraineurs without aura are hypersensitive to external visual and auditory stimuli. Furthermore, our study offers a new quantitative and objective method for evaluating the hypersensitivity of patients with migraine to sound and light.

## Competing interests

The authors declare that they have no competing interests.

## Authors’ contributions

Conceived and designed the experiments: WY, JY, and JW. Recruited and diagnosed the patients: BC and SY. Performed the experiments: WY, BC, YY, and SY. Analyzed the data: WY, BC, JY, JW, and SY. Wrote the paper: WY, JY, JW, and SY. All authors read and approved the final manuscript.
